# Membranes from Carboxymethyl Cellulose/Carboxylated Graphene Oxide for Sustainable Water Treatment by Pervaporation and Nanofiltration

**DOI:** 10.3390/molecules30183751

**Published:** 2025-09-15

**Authors:** Mariia Dmitrenko, Olga Mikhailovskaya, Anna Kuzminova, Anton Mazur, Rongxin Su, Anastasia Penkova

**Affiliations:** 1 St. Petersburg State University, 7/9 Universitetskaya nab., St. Petersburg 199034, Russia; st113220@student.spbu.ru (O.M.); a.kuzminova@spbu.ru (A.K.); a.mazur@spbu.ru (A.M.); 2 State Key Laboratory of Chemical Engineering, School of Chemical Engineering and Technology, Tianjin University, Tianjin 300072, China; surx@tju.edu.cn

**Keywords:** carboxymethyl cellulose, carboxylated graphene oxide, pervaporation, nanofiltration, wastewater treatment

## Abstract

Developing efficient bio-based membranes is key to sustainable wastewater treatment, especially when they can be applied across multiple separation processes for components of varying molecular weights. This study reports the development and characterization of bio-based mixed matrix membranes from carboxymethyl cellulose (CMC) modified with synthesized carboxylated graphene oxide (GOCOOH), aimed at improving performance in both pervaporation and nanofiltration for water treatment. Membrane design was optimized by adjusting the GOCOOH content, applying chemical cross-linking (by immersing in glutaraldehyde with H_2_SO_4_), and developing highly effective supported membranes (by the deposition of a thin selective CMC-based layer onto a porous substrate). Comprehensive characterization was performed using spectroscopic, microscopic, and thermogravimetric analyses and contact angle measurements. The optimized cross-linked supported CMC/GOCOOH (5%) membrane demonstrated significantly improved transport properties: a 2.5-fold increased permeation flux and over 99.9 wt.% water in permeate in pervaporation dehydration of isopropanol, and high rejection rates—above 98.5% for anionic dyes and over 99.8% for heavy metal ions in nanofiltration. These findings demonstrate that CMC/GOCOOH membranes are promising, sustainable materials suitable for multiple separation processes involving components of varying molecular weights, contributing to more efficient and eco-friendly wastewater treatment solutions.

## 1. Introduction

In the face of growing environmental challenges, there is an urgent need to develop sustainable technologies that balance industrial performance with ecological responsibility. As society increasingly demands higher standards for product purity, resource efficiency, and environmental protection, the development of advanced separation technologies becomes a critical priority [1,2]. Membrane technologies stand out as a key solution, offering energy-efficient, environmentally friendly, and scalable alternatives to traditional separation methods, with advantages such as low chemical consumption, operational simplicity, and reduced environmental footprint [3,4]. However, the development of next-generation membrane materials remains essential, particularly those derived from renewable resources. Biopolymer-based membranes offer a promising path forward, combining sustainability with tunable functionality [5]. Their potential becomes especially significant when engineered for dual applications—capable of operating across multiple membrane processes such as pervaporation and nanofiltration—and for separating a broad range of substances with varying molecular weights. Pervaporation is commonly used to separate mixtures containing low-molecular-weight compounds, especially for water purification from organic solvents [6]. In contrast, nanofiltration offers an energy-efficient method for water treatment by removing contaminants such as dyes and heavy metals, presenting a viable, lower-energy alternative to traditional reverse osmosis [7].

Carboxymethyl cellulose (CMC) is a widely studied water-soluble derivative of cellulose, recognized for its natural abundance, environmental friendliness, and excellent biodegradability, making it an attractive candidate for sustainable material development [8,9]. Owing to its favorable properties—such as mechanical strength, surface hydrophilicity, and low cost—CMC and its hybrid materials are widely applied across various industrial fields [10]. In membrane technologies, CMC has been effectively used as a membrane material for diffusion (pervaporation and gas separation [8,11,12,13]) and one of the pressure-driven processes, nanofiltration [14]. It should be noted that in most reported studies, CMC is used to form polyelectrolyte complexes with reagents such as poly(2-methacryloyloxy ethyl trimethylammonium chloride [15], poly(diallyldimethylammonium chloride) [16], gelatin [12], chitosan [17], which are then used to develop membranes with improved separation performance and mechanical properties. However, to the best of our knowledge, there are limited studies on the use of pristine CMC as a matrix of mixed matrix membranes (MMMs) for pervaporation [13,18,19,20,21], pervaporation catalytic membrane reactor [22], and nanofiltration [23,24].

The design of MMMs using carefully selected biopolymers and functional modifiers is crucial to overcoming the conventional trade-off between selectivity and permeability [25]. This approach enables the creation of high-performance membranes tailored to meet the stringent demands of modern wastewater treatment, making biopolymer-based MMMs an increasingly vital area of research and innovation. Carbon nanoparticles have emerged as highly promising modifiers for MMMs, with graphene oxide (GO) standing out due to its exceptional properties [26]. GO features a high specific surface area, strong hydrophilicity, biocompatibility, low toxicity, excellent mechanical strength, and abundant oxygen-containing functional groups, which facilitate its uniform dispersion in polar polymer matrices [27]. Numerous studies have explored composites with GO and its modified forms as effective adsorbents [28,29,30,31]. In particular, adsorbent from CMC microbeads modified with carboxylated graphene oxide (GOCOOH) was studied for the adsorptive removal of cationic methylene blue dye in the work [32]. However, the challenge of recovering these adsorbents after treatment limits their standalone use. In this study, carboxylated GO was synthesized and for the first time incorporated as a modifier to investigate its influence on the structural, physicochemical, and transport properties of CMC-based MMMs. The additional carboxyl groups in GOCOOH enhance its compatibility and dispersion within the polymer matrix and enable diverse modifications via ionic, covalent, and hydrogen bonding interactions, thereby improving membrane performance [33].

Thus, the aim of this study was to develop and study novel bio-based MMMs from CMC modified with GOCOOH for enhanced sustainable water treatment by dual application in both pervaporation and nanofiltration. The membrane formulation was refined by adjusting the amount of modifying agents, chemical cross-linking techniques, and creating high-performance supported membranes featuring a thin selective layer based on CMC, which was applied to a specially developed porous substrate. The resulting membranes were examined using a range of analytical methods, including spectroscopic methods (Fourier transform infrared (FTIR) and nuclear magnetic resonance (NMR) spectroscopy), microscopic methods (scanning electron (SEM) and atomic force (AFM) microscopy), thermogravimetric analysis (TGA), and contact angle evaluation. Both dense and supported CMC-based membranes were assessed for their transport behavior in the pervaporation-based dehydration of isopropanol. Additionally, the optimized supported membrane was tested for its performance in nanofiltration of aqueous solutions containing anionic dyes, heavy metal ions, and salts of magnesium and sodium.

## 2. Results and Discussion

This section is divided into two main subsections. Section 2.1 presents the investigation of changes in the structural and physicochemical properties of the membranes during the modification process using various analytical methods. Section 2.2 focuses on optimizing CMC-based membranes for enhanced pervaporation performance by selecting the optimal GOCOOH content in the matrix, cross-linking, and developing highly effective supported membranes (Section 2.2.1). It also includes the evaluation of transport parameters of MMMs based on CMC/GOCOOH composite with optimal properties in nanofiltration for advanced water treatment from dyes, heavy metal ions and salts (Section 2.2.2). 

### 2.1. Structure and Physicochemical Properties of CMC-Based Membranes

The composition of the developed membranes and the parameters of their synthesis—including details on the GOCOOH content and the use of the porous polyphenylene isophthalamide (PA) substrate for supported membranes (indicated by a hyphen and a slash, respectively), as well as the notation of the cross-linking agent glutaraldehyde (GA) as a superscript—are summarized in Table 1.

**Figure 1:**
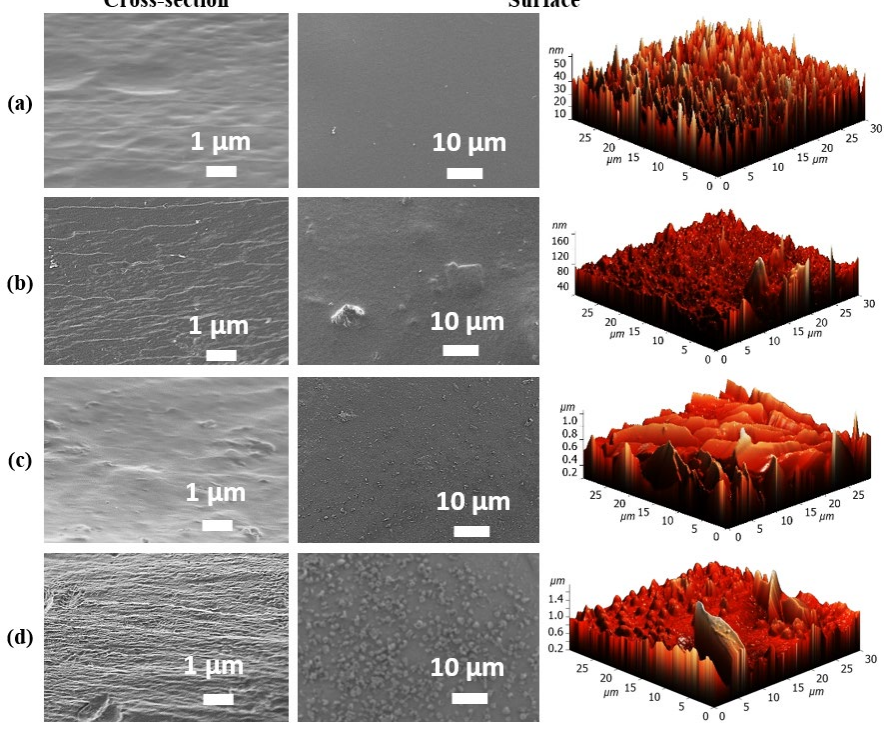
SEM and AFM images of dense (**a**) CMC, (**b**) CMC-5, (**c**) CMC^GA^ and (**d**) CMC-5^GA^ membranes.

The CMC-based membrane morphology was studied using microscopic methods. SEM and AFM images of the surface and cross-section for dense untreated and cross-linked membranes are shown in Figure 1.

For the pristine CMC membrane, a uniform, wide, folded linear structure was observed in cross-section, along with a smooth, consistent surface free of any noticeable defects or irregularities [18]. The incorporation of GOCOOH into the CMC matrix resulted in a more folded and rougher cross-sectional morphology and surface characterized by prominent GO sheets forming rounded folds (as cloud-like formations) in the CMC-5 membrane, attributed to the presence of the filler [32,34]. This effect was also noticed when introducing other carbon nanoparticles into the polymer matrix [16,35]. Moreover, cross-linking with GA led to a more pronounced rough morphology in the cross-linked membranes, featuring distinct folds in cross-sections and a uniform distribution of the modifier on the surface. This structural roughness likely increases the membrane’s surface area, enhancing contact with the separated components [36]. Additionally, to determine elemental composition and to demonstrate a uniform distribution of the carbon signal throughout membranes, the chemical analysis of membranes (CMC, CMC-5 and CM-5^GA^) was carried out by energy dispersive X-ray spectroscopy (EDS) (Figure S1).

**Figure 2:**
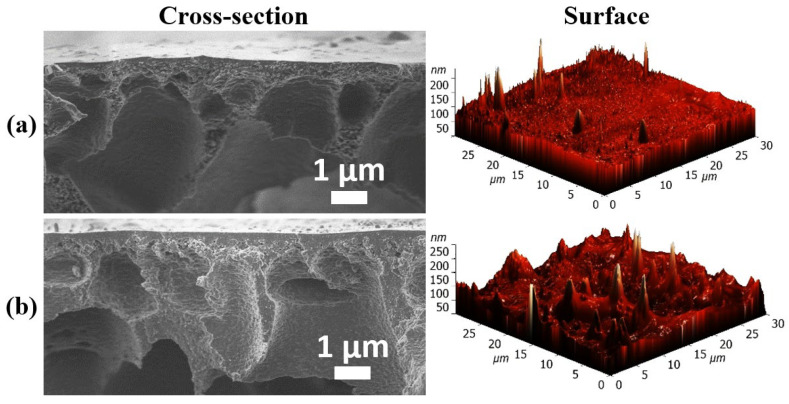
SEM cross-sectional and AFM surface images of supported (**a**) CMC/PA^GA^ and (**b**) CMC-5/PA^GA^ membranes.

SEM and AFM images of the surface and cross-section for cross-linked supported membranes are shown in Figure 2. The surface SEM images for these membranes were identical to those of the dense membranes.

Cross-sectional SEM images of the supported membranes clearly reveal two distinct regions: (1) an upper dense, non-porous selective layer, approximately 300 ± 50 nm thick, and (2) a lower porous PA substrate that provides mechanical support to the thin upper layer. Notably, the use of the PA substrate enabled the formation of the thinnest CMC-based selective layer compared to other substrates such as polyacrylonitrile [16] and cellulose acetate [24], which significantly contributes to the high performance and productivity of the fabricated membranes.

According to the pervaporation mechanism, the sorption of components onto the membrane surface is the first stage. Therefore, modifications that alter the surface characteristics are likely to significantly influence the membrane’s separation performance. To assess these changes, surface roughness parameters were determined from AFM images, and the water contact angles of membranes were measured (Table 2).

The data on roughness and contact angle for the CMC membrane corresponded to the values obtained previously in the works [18,23]. For all modified membranes compared to pristine CMC-based membranes, the introduction of GOCOOH into polymer matrix led to hydrophilization of the membrane surface (decrease in contact angle value [37]) due to its abundant functional groups [32] and an increase in its roughness, which is in agreement with the SEM images (Figure 1). The CMC^GA^ and CMC-5^GA^ membranes had the highest surface roughness due to the cross-linking process, resulting in a more complex and uneven structure at the molecular level [38]. Meanwhile, for cross-linked supported membranes (CMC/PA^GA^ and CMC-5/PA^GA^), the roughness decreases compared to cross-linked dense membranes due to the influence of the PA substrate on the deposited thin selective layer, and the values of the contact angles of cross-linked dense and supported membranes at the same level indicate uniform and long-term formation of a layer from CMC and its composite without defects.

**Figure 3:**
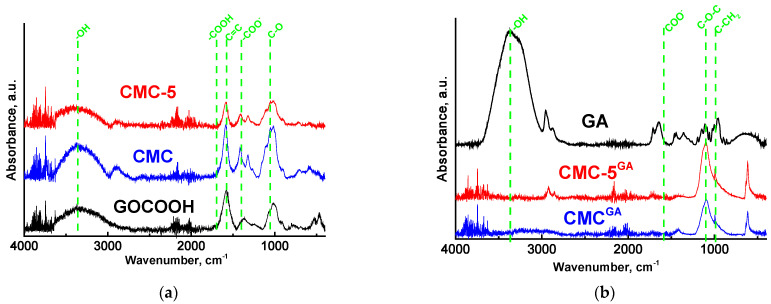
FTIR spectra of (**a**) GOCOOH, untreated CMC-based membranes and (**b**) GA, cross-linked CMC-based membranes.

The CMC-based membranes were analyzed using FTIR and NMR spectroscopy to evaluate structural changes resulting from modification, including the introduction of GOCOOH and cross-linking. The FTIR spectra of pristine GOCOOH, GA and the dense membranes are shown in Figure 3.

The successful formation of GOCOOH was verified by the emergence of peaks at 1700 cm^−1^ (–COOH functional groups) and 1058 cm^−1^ (C–O functional groups) and by the shift in the peak associated with the C=C bond to 1577 cm^−1^ [32]. This shift can be attributed to the high concentration of carbonyl groups in GOCOOH [39]. 

The FTIR spectrum obtained for the CMC membrane reveals several distinct spectral features. Notably, there are pronounced peaks at 3366 cm^−1^, corresponding to –OH functional groups [40], at 1585 cm^−1^, associated with COO^−^ groups, and at 1414 cm^−1^, indicative of the carboxylate groups in their salt form [41]. Furthermore, a strong absorption band is evident in the range of 900–1250 cm^−1^, which signifies the presence of ether linkages and bonding patterns characteristic of the polymer structure [41]. GOCOOH was incorporated into the polymer matrix through physical interactions, specifically hydrogen bonding between the carboxylic groups of CMC and the hydroxyl groups on the GO sheets [32,34,42]. This interaction is evidenced by the notable reduction in peak intensities at 3372 and 1584 cm^−1^ observed in the FTIR spectrum of the CMC-5 membrane.

For the CMC^GA^ membrane, a new peak appearing at 1097 cm^−1^ indicates the ether (C–O–C) linkages. The absorption bands originally observed at 3366 and 1585 cm^−1^ in the CMC membrane show a downshift after cross-linking, while the intensity of the band at near 1700 cm^−1^ increases. In addition, after cross-linking, a new band is seen at 994 cm^−1^ (C-CH_2_). These spectral changes confirm that GA cross-linking occurs through a chemical mechanism: a reaction between CMC and GA, leading to the formation of covalent bonds [43,44]. The potential pathway of CMC cross-linking with GA is detailed in [45,46]. Notably, these structural modifications are even more pronounced in the modified cross-linked CMC-5^GA^ membrane, suggesting that GOCOOH also actively participates in the cross-linking reaction with GA.

**Figure 4:**
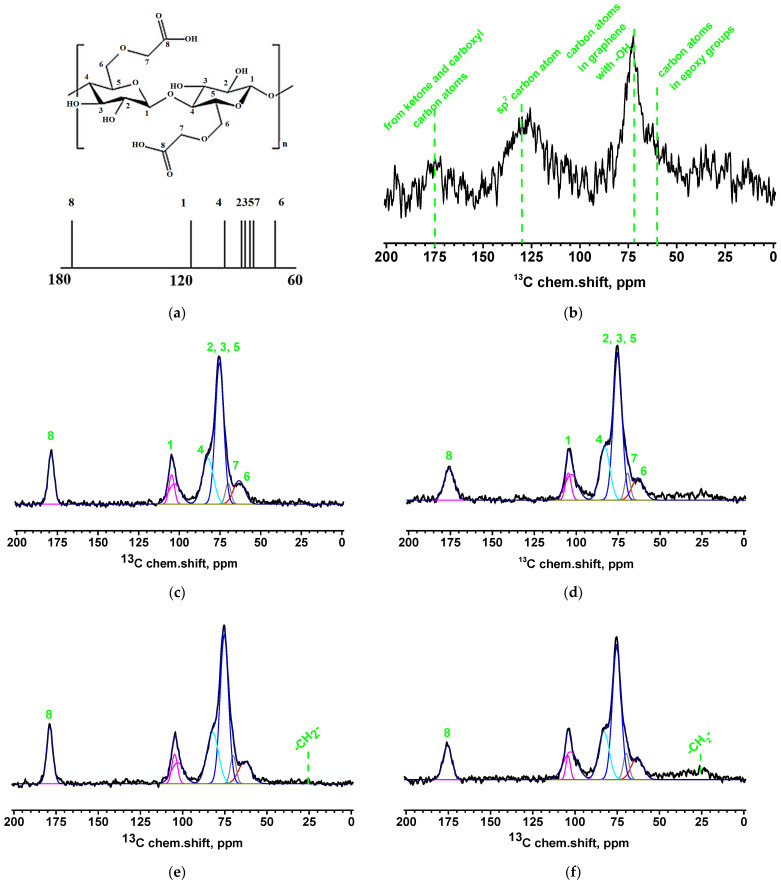
^13^C NMR spectra of (**a**) the CMC polymer block, (**b**) GOCOOH, (**c**) CMC, (**d**) CMC-5, (**e**) CMC^GA^, and (**f**) CMC-5^GA^ membranes. The numbers indicate the numbered positions of carbon atoms.

Figure 4 presents the NMR spectra of GOCOOH and the developed CMC-based membranes, along with the structural scheme of the CMC polymer block showing numbered carbon atom positions and schematic spectra with corresponding relative peaks (Figure 4a) [47,48].

The NMR spectrum of GOCOOH exhibits a peak with a maximum around 130 ppm, corresponding to the sp^2^ carbon atoms of the graphene framework (Figure 4b). A weak, broad peak near 175 ppm is attributed to overlapping signals from ketone and carboxyl carbon atoms [49,50]. The peak at approximately 70 ppm corresponds to carbon atoms in the graphene structure bearing hydroxyl groups, while the broad, unresolved peak around 60 ppm is associated with carbon atoms in epoxy groups [49].

Figure 4c,d show the spectra of the CMC and CMC-5 membranes. These spectra display distinct peaks corresponding to the carbon atoms of carboxyl groups (around 178 ppm). A separate peak is observed for the carbon atoms at position 1, consisting of two unresolved signals attributed to crystalline (104.7 ppm) and amorphous (102.9 ppm) cellulose [51]. Additionally, a non-uniformly broadened peak with a maximum at approximately 75.3 ppm is present. This peak can be deconvoluted into four components: a peak at ~82.5 ppm corresponding to carbon atoms at position 4; an intense peak near 75.3 ppm associated with overlapping signals from carbon atoms at positions 2, 3, and 5; a weak shoulder near 70 ppm corresponding to carbon atoms at position 7; and a broad, low-intensity signal around 63.2 ppm, which corresponds to carbon atoms at position 6. The addition of GOCOOH into the CMC matrix does not significantly alter the spectral characteristics of membranes. This suggests that the modifying additive affects membrane properties primarily through mechanical contributions (hydrogen bonding confirmed by FTIR, Figure 3) rather than changes in chemical structure.

For the spectra of CMC^GA^ and CMC-5^GA^ (Figure 4e,f), it is noticeable that the signal corresponding to the carbon atoms at position 8 shifts toward lower chemical shift values and becomes broader. This is most likely due to the involvement of cellulose groups in the chemical cross-linking of the membrane structure. Furthermore, analysis of the peak ratio between crystalline and amorphous cellulose indicates a reduction in the crystalline phase upon modification—from 39% in CMC to 38%, 32%, and 21% in CMC-5, CMC^GA^, and CMC-5^GA^, respectively. It is also worth noting that the peak width of the carboxyl group signal in the CMC-5^GA^ membrane (2.6 ppm) is narrower than that in the CMC^GA^ membrane (3.1 ppm), which may suggest that the addition of GOCOOH enhances homogeneity in the cross-linking region. Additionally, in the spectra of cross-linked membranes, a weak broad signal near 25 ppm—at the level of background noise—can be observed. This signal corresponds to the –CH_2_– groups of GA. 

The thermal stability of the developed dense membranes was assessed by TGA. The corresponding thermograms (TG) are shown in Figure S2 of Supplementary Materials. The TG reveal three distinct stages of weight loss [18]. The initial stage, occurring up to approximately 200 °C, corresponds to the evaporation of water molecules bound within the membrane matrix. The second stage, extending to around 300 °C, is associated with the degradation of functional groups and the release of carbon dioxide from the polymer backbone. The final stage, occurring above 300 °C, reflects the breakdown of the CMC’s structural framework [52]. In contrast, the TG curves of the modified membranes exhibit reduced weight loss below 300 °C, which suggests slightly enhanced thermal stability—likely due to the incorporation of GOCOOH within the polymer matrix [32]. This may be due to the strengthened interfacial interactions between membrane components (confirmed by FTIR, Figure 3), which hinder the mobility of the CMC chains and restrict their movement (confirmed by NMR, Figure 4) and decomposition of the membrane at elevated temperatures [53,54].

### 2.2. Transport Characteristics of CMC-Based Membranes

#### 2.2.1. Pervaporation Performance

**Figure 5:**
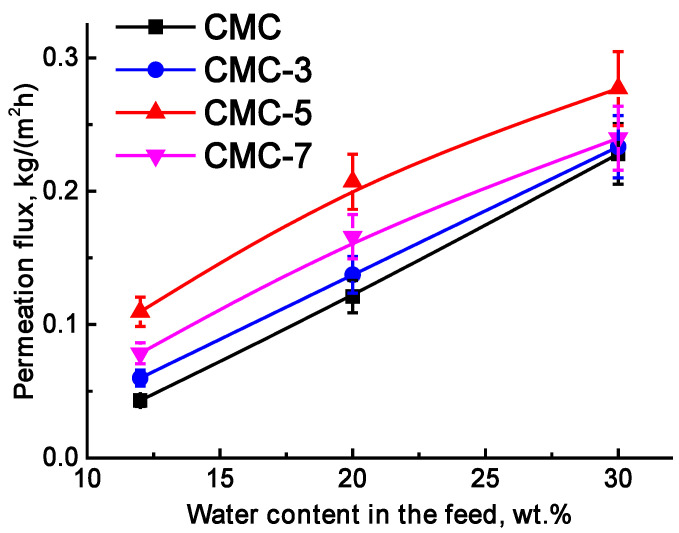
Dependence of permeation flux on water content in the feed for dense CMC and CMC/GOCOOH membranes in pervaporation dehydration of isopropanol (12–30 wt.%).

To optimize the membrane composition, GOCOOH nanoparticles were introduced into the CMC matrix in different concentrations (3–7 wt.%) to select the optimal modifier content. The membranes were evaluated in pervaporation dehydration of isopropanol (12–30 wt.% water) (Figure 5).

**Figure 6:**
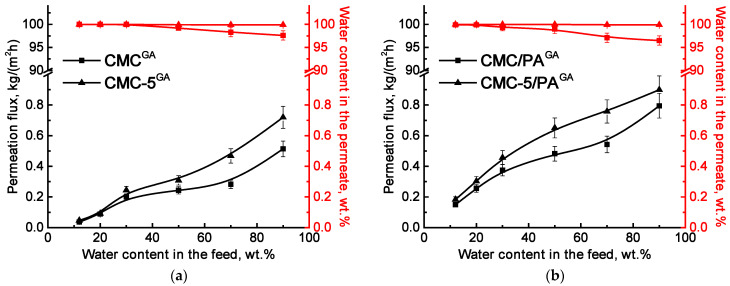
Dependence of permeation flux and water content in the permeate on water content in the feed for cross-linked (**a**) dense and (**b**) supported CMC-based membranes in pervaporation dehydration of isopropanol (12–90 wt.%).

All membranes demonstrated excellent water selectivity, achieving 99.9 wt.% water content in the permeate across mixtures with varying compositions. The addition of nanoparticles to the CMC resulted in a notable improvement in permeation flux. This improvement is linked to increased surface and internal roughness (evidenced by SEM and AFM, Figure 1), which arose from hydrogen bonding between the polymer and the incorporated modifier (confirmed by FTIR and NMR analysis, Figure 3 and Figure 4). Contact angle measurements (Table 2) also indicated enhanced surface hydrophilicity, attributed to the abundance of oxygen-containing functional groups in GOCOOH. These GOCOOH nanoparticles not only increased hydrophilicity but also served as selective pathways for water transport [37,55,56]. However, the CMC-7 membrane showed reduced permeation flux compared to CMC-5, likely due to nanoparticle aggregation reducing effective permeability. Such aggregation may have created impermeable regions, hindering component transport through the membrane [37,56]. Consequently, a GOCOOH concentration of 5 wt.% in the CMC matrix was identified as optimal, as it resulted in the CMC-5 membrane achieving the highest permeation flux among all tested membranes—an increase of over 22% compared to the pristine CMC membrane. The CMC and CMC-5 membranes were then cross-linked with GA to increase their stability and suitability for separating mixtures with high water content (Figure 6a). 

The cross-linked membranes demonstrated high stability and selectivity in the pervaporation dehydration of isopropanol across the entire concentration range. Cross-linking resulted in a permeation flux comparable to that of the untreated membranes. This behavior could be attributed to structural changes—specifically, the formation of a rougher and more hydrophilic surface (as confirmed by SEM, AFM, and contact angle data, Figure 1 and Table 2), along with a reduction in the free volume between polymer chains during cross-linking [57]. In the case of the CMC-5^GA^ membrane, not only was an increase in permeation flux observed, but also an improved water content in the permeate (above 99.9 wt.%) compared to the CMC^GA^ membrane, where the water content decreased to 97.6 wt.%. These improvements are the result of the synergistic effect of the GOCOOH modifier and the GA cross-linking agent on the structure and properties of the membrane matrix, in particular, the simultaneous modification with GOCOOH and GA cross-linking leads to a reduction in the crystalline phase of the CMC matrix, while the presence of this modifier enhances homogeneity within the cross-inking region (confirmed by NMR, Figure 4).

For potential industrial applications in water purification from low molecular weight substances (e.g., toxic solvents), cross-linked supported membranes were developed by applying a thin layer based on CMC and its composite optimal CMC/GOCOOH (5%) onto a porous PA substrate. This approach enabled a two-fold increase in permeation flux (0.15–0.75 kg/(m^2^h) for CMC/PA^GA^, 0.18–0.90 kg/(m^2^h) for the CMC-5/PA^GA^) compared to dense membranes while maintaining high selectivity for water. For example, the permeation flux of the dense CMC-5^GA^ membrane is 0.244 kg/(m^2^h), while for the supported CMC-5/PA^GA^ membrane it increased to 0.457 kg/(m^2^h) when separating an isopropanol/water mixture (70/30 wt.%). For the CMC/PA^GA^ membrane, a slight decrease in water content in the permeate was observed (above 96 wt.%), likely due to excessive swelling of a thin selective layer with thickness of 300 nm (confirmed by SEM, Figure 2) in the separated mixture [58]. In contrast, the modified CMC-5/PA^GA^ membrane maintained high selectivity (above 99.9 wt.%), attributed to the presence of GOCOOH. Thus, the developed CMC-5/PA^GA^ membrane exhibited the best performance for the pervaporation dehydration of isopropanol, owing to its improved stability in dilute mixtures, a permeation flux exceeding 2.5, and the ability to maintain high selectivity (above 99.9 wt.%) compared to the untreated pristine CMC membrane.

#### 2.2.2. Nanofiltration Performance

**Figure 7:**
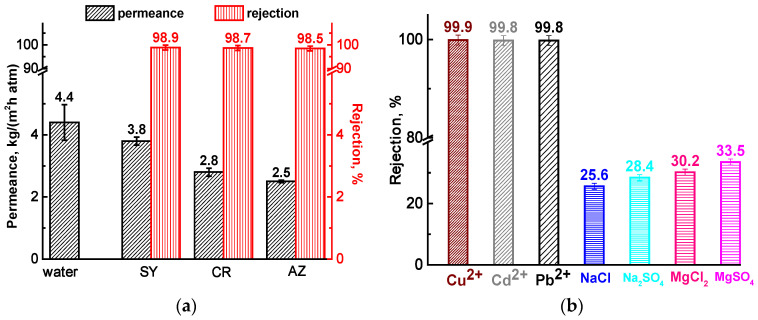
Transport parameters of the CMC-5/PA^GA^ membrane in nanofiltration of (**a**) water and dye solutions (Sunset Yellow (SY), Congo Red (CR), and Alphazurine (AZ)), (**b**) heavy metal ion and salt solutions.

For the prospective dual application of the developed membrane, the optimized CMC-5/PA^GA^ membrane was also evaluated in nanofiltration of aqueous solutions of anionic dyes Sunset Yellow (SY), Congo Red (CR), and Alphazurine (AZ), heavy metal ions (Cu^2+^, Cd^2+^, Pb^2+^), Mg and Na salts (Figure 7). The membrane permeance during nanofiltration of aqueous solutions containing heavy metal ions and salts is not shown, as it was equivalent to that of pure water.

The permeance of the CMC-5/PA^GA^ membrane depends on the molecular weight of the dyes (Figure 7a), with permeance decreasing as molecular weight increases. The addition of modifier particles and cross-linking with GA improved membrane surface roughness (as shown by AFM, Table 2), enhanced surface hydrophilicity (confirmed by contact angle measurements, Table 2), and created selective water channels [37], all contributing to increased permeance. Dye rejection is influenced by multiple mechanisms, including molecular sieving, differences in diffusion and solubility, and the Donnan effect [59,60], with electrostatic interactions playing a dominant role. The CMC-5/PA^GA^ membrane achieved high dye rejection (above 98.5%) due to the electrostatic repulsion of anionic dyes by the negatively charged surface of the membrane, primarily from oxygen-containing groups on the GOCOOH [61]. To evaluate the resistance of membrane to dye contamination, the FRR parameter was calculated, which was equal to 78% after SY penetration, 75% after CR penetration and 72% after AZ penetration. Thus, it was shown that membranes are subject to fouling due to the adsorption of dyes on their surface. Additionally, the modifier could act as a dye adsorbent, further enhancing rejection efficiency [62]. 

The CMC-5/PA^GA^ membrane exhibited a high rejection rate for heavy metal ions, exceeding 99.8% (Figure 7b). This value surpasses those reported for previously developed CMC-based membranes supported on cellulose acetate and modified with various Zn-based metal–organic frameworks [23]. The optimal membrane from CMC/Zn(BDC)Si (15%) composite had rejection coefficients of 97% Cu^2+^, 94% Cd^2+^, 97% Pb^2+^. This enhanced performance is likely due to the presence of GOCOOH, which has been shown to effectively reject heavy metal ions [63,64]. The negatively charged surface of the CMC-5/PA^GA^ membrane, resulting from CMC and GOCOOH modification, promotes electrostatic interactions with positively charged metal ions, facilitating their adsorption and improving rejection efficiency [65]. However, it should be noted that nanofiltration effectiveness of these membranes in real-world water treatment (with complex mixtures) would be hindered by factors like fouling, degradation (particularly due to chemicals such as acids), reduced rejection, and the unpredictability of operating conditions (changing pH and temperature) [66,67]. Single-solute heavy metal ion model solutions do not replicate the complex mixture of dissolved and suspended substances present in real water, which play a role in membrane fouling, degradation, and other performance problems [68].

Salt rejection for the CMC-5/PA^GA^ membrane followed the order: MgSO_4_ > MgCl_2_ > Na_2_SO_4_ > NaCl (Figure 7b). This trend aligns with the well-established understanding that charge effects dominate nanofiltration performance for low-concentration inorganic electrolytes [69]. The membrane’s active layer, containing carboxymethyl groups, exhibits stronger electrostatic repulsion toward sulfate ions (SO_4_^2−^) compared to chloride ions (Cl^−^). Additionally, the lower rejection of Na salts compared to Mg salts may result from GOCOOH high affinity for Mg^2^⁺ ions [70], due to their higher charge density. Similar low salt rejection trends for divalent cations in CMC-based membranes have also been reported in the literature [71].

Thus, the optimized cross-linked supported CMC-5/PA^GA^ membrane demonstrated significantly enhanced separation performance—achieving a 2.5-fold increase in permeation flux and over 99.9 wt.% water in the permeate in pervaporation dehydration of isopropanol, along with high nanofiltration rejection for anionic dyes (>98.5%) and heavy metal ions (>99.8%)—highlighting its potential as a sustainable, multifunctional material for efficient and eco-friendly wastewater treatment.

## 3. Materials and Methods

### 3.1. Materials

Carboxymethyl cellulose (CMC, 400 kDa, a Brookfield viscosity of 7468 mPa·s at 25 °C for a 1% aqueous solution), sourced from Bioprod LLC (St. Petersburg, Russia), was used as a membrane matrix. Graphene oxide (GO) nanoparticles from Fullerene Technologies (St. Petersburg, Russia) were utilized as a modifying agent to create mixed matrix membranes (MMMs). The preparation of carboxylated GO (GOCOOH) involved sodium hydroxide (NaOH, Sigma-Aldrich, St. Louis, MO, USA), chloroacetic acid (ClCH_2_COOH, LenReactive, St. Petersburg, Russia), and methanol (MeOH, 99.5 wt.%, Vekton, St. Petersburg, Russia). Polyphenylene isophthalamide (PA, Fenylon C2, batch 12/2018) was obtained from UNIPLAST Ltd. (Vladimir, Russia) served as the polymer for fabricating substrates for supported CMC-based membranes. The solvent N,N-dimethylacetamide (DMAc) from Vekton (St. Petersburg, Russia), glutaraldehyde (GA, Sigma-Aldrich, St. Louis, MO, USA), sulfuric acid (H_2_SO_4_), and isopropanol (iPrOH) from Vecton (St. Petersburg, Russia) were used without treatment. 

### 3.2. Preparation of Carboxylated GO

Carboxylated GO was synthesized as follows [32]: to a suspension of GO (0.25 g in 100 mL of distilled water), 5 g of NaOH and 5 g of ClCH_2_COOH were added. The mixture was then ultrasonicated to promote the reaction: ultrasound for 30 min, followed by 30 min of rest, repeated every hour for a total of 6 h. After synthesis, the GOCOOH was neutralized and washed successively with distilled water and methanol to remove any residual reactants. Finally, the purified carboxylated GO was dried at 50 °C for 12 h to obtain the final modified nanoparticles.

### 3.3. Membrane Preparation

#### 3.3.1. Dense Membranes

The CMC-based membranes were fabricated as follows: a 1 wt.% polymer solution was prepared by dissolving CMC in distilled water under continuous stirring at 45 °C for 4 h. The modification was carried out by the introduction of aqueous GOCOOH dispersions (20 g/L) into the CMC solution at concentrations of 3, 5 and 7 wt.% by weight of polymer. Then it was homogenized under stirring for 2 h followed by ultrasonication (LLC “MagazinLAB”, St. Petersburg, Russia) at ambient temperature for 1 h. Dense (nonporous) membranes based on CMC and CMC/GOCOOH composites were formed by solvent evaporation on Petri dishes in an oven (Ekros Co., St. Petersburg, Russia) at 40 °C for 24 h (Figure S3). The resulting membranes had a uniform thickness of 50 ± 5 μm, confirmed by micrometer measurements.

#### 3.3.2. Supported Membranes

The supported membranes were fabricated via physical adsorption by depositing a thin, nonporous layer based on CMC or CMC/GOCOOH (5 wt.%) composite (1 wt.% solution), prepared similarly to the formulations used for dense membranes, onto a porous substrate from PA prepared via non-solvent induced phase separation (NIPS) [72] (Figure S4). A specified amount of PA powder was dissolved in DMAc at 110 °C under stirring for 4 h, resulting in a 15 wt.% polymer solution. The solution was then placed in an ultrasonic bath for degassing. It was subsequently cast onto a glass plate using a casting blade with a slit width of 200 μm, before being immersed in a coagulation bath with distilled water at room temperature for 24 h to allow solvent residues to diffuse into the bath. Then the PA substrate was secured onto a hollow steel ring, and the CMC solution or composite was poured onto the substrate from the ring’s side. Any excess casting solution was then removed. After deposition, the supported membranes were left at ambient temperature for 24 h to allow solvent evaporation, resulting in the formation of a dense (nonporous), thin selective CMC or CMC/GOCOOH-based layers on the PA substrate.

#### 3.3.3. Cross-Linking of Membranes

To ensure membrane stability in dilute media, the dense and supported membranes based on CMC and CMC/GOCOOH (5%) composite were chemically cross-linked: membranes were immersed in a solution of isopropanol and water (90/10 *v*/*v*) containing 1 wt.% GA with the addition of 0.5 wt.% H_2_SO_4_ as a catalytic component for 5 min at ambient temperature [37]. After treatment, the samples without washing with water were dried in an oven at 40 °C for 5 min.

### 3.4. Membrane Performance Investigation

#### 3.4.1. Pervaporation

The membranes were investigated in pervaporation dehydration of isopropanol (12-90 wt.% water) at an ambient temperature in a cell with an effective area of 9.6 cm^2^ operating in a stationary mode with constant stirring (Figure S5). To maintain a stable composition of the feed and to ensure reproducibility of membrane transport characteristics, 100 g of feed solution was used. Prior to the measurements, membranes were pre-swollen: the first portion of permeate collected during 30 min before reaching the equilibrium state was excluded from the analysis. At least 0.3 g of permeate sample was required to reliably determine the composition using a gas chromatograph Chromatek Crystal 5000.2 (“Chromatek”, Yoshkar-Ola, Russia). Introduction of samples of analyzed liquids into the chromatograph was carried out automatically using a dosing device “DAZH-2M” (“Chromatek”, Yoshkar-Ola, Russia).

To ensure reliability, each pervaporation experiment was conducted a minimum of three times using the same membrane sample, and the average values were reported. The approximate measurement accuracy for the transport parameters was as follows: below 1% for water concentration in the permeate, ±10% for permeation flux in dense membranes, and ±5% for permeation flux in supported membranes. Standard deviations for all transport values are displayed in the corresponding figures.

The permeation flux (*J*) was calculated using Equation (1) [73]:

*J* = *W*/(*A*·*t*), (1)

where *A* denotes the surface area of the membrane (m^2^), *t* is the duration of the permeation process (h), and *W* refers to the total mass of the permeated substances (kg).

#### 3.4.2. Nanofiltration

Nanofiltration was performed using a dead-end cell (effective area = 0.002 m^2^) under ambient temperature and pressures ≤ 50 atm (Figure S6). To ensure constant concentration and avoid polarization, experiments involved the alternate passage of water and the separated mixture with a volume of 500 mL while stirring vigorously. Each membrane underwent at least one week of testing, bracketed by water permeation runs to confirm property stability. To minimize errors, each nanofiltration measurement was carried out for 3 days and at least three times for one membrane sample, and the results were averaged. The mean accuracy of the transport parameters was as follows: less than 1% for rejection and less than ±10% for permeance. All standard deviations of transport parameters are presented in the figures.

Membrane permeance (*L*) was calculated according to Equation (2) [73]:

*L* = *m*/(*A*·*t*∙*∆P*) (2)

where *m* (kg) is the permeate mass, *t* (h) is the collection time, *A* (m^2^) is the effective membrane area, and *ΔP* (atm) is the transmembrane pressure.

The rejection (*R*) was calculated according to Equation (3):

(3) $$R=\left(1-\frac{C_{perm}}{C_{feed}}\right)\times 100$$

where *C*_perm_ and *C*_feed_ are the concentrations of the component(s) in the permeate and feed, respectively.

The flux recovery ratio (*FRR*) was calculated according to Equation (4):

(4) $$FRR=\frac{L}{L_{w}}\times 100$$

where *L* is the flux of water after the contaminant permeation, and *L_w_* is the pristine water flux.

The nanofiltration efficacy of CMC-based membranes was tested in separating dyes from water. The feed comprised 10 mg/L aqueous solutions of the anionic dyes Sunset Yellow (SY), Congo Red (CR), and Alphazurine (AZ), selected for their molecular weight range (Table S1). Concentration analysis was performed using a PE-5400UV spectrophotometer (EKROSKHIM Co., St. Petersburg, Russia) at the absorption maxima—483 nm for SY, 505 nm for CR, and 628 nm for AZ.

CMC-based membranes with optimized properties were also tested using 50 mg/L aqueous solutions of Cu(NO_3_)_2_, Pb(NO_3_)_2_, and Cd(NO_3_)_2_ [23] and Na and Mg salts (NaCl, Na_2_SO_4_, MgCl_2_, MgSO_4_ salts with a concentration of 5 mM). After each experiment with heavy metal ions, the cell was rinsed with a 5 g/L aqueous Trilon B solution. Metal ion concentrations in the feed and permeate were quantified by stripping voltammetry (TA-4 voltammetric analyzer, Tomanalit, Russia) using a mercury film working electrode and silver chloride reference/auxiliary electrodes. Na and Mg salt content in feed and permeate was investigated using conductivity meter Cond 7110 from “Xylem Analytics Germany GmbH” (Weilheim, Germany). Measurements were carried out by conductivity with further conversion to concentration.

### 3.5. Structure and Properties Investigation

The structural characteristics of the synthesized CMC-based membranes were examined using Fourier Transform Infrared (FTIR) spectroscopy. Spectral data were collected with an IRAffinity-1S spectrometer (Shimadzu, Kyoto, Japan) equipped with an attenuated total reflection (ATR) accessory (PIKE Technologies, Madison, WI, USA), operating in the 450–4000 cm^−1^ range at a temperature of 25 °C. To further investigate the molecular structure, nuclear magnetic resonance (NMR) spectroscopy was employed for dense CMC-based membranes using a Bruker Avance III 400 WB spectrometer (Bruker, Billerica, MA, USA) with a 9.4 T magnetic field and a 4 mm magic angle spinning (MAS) probe. Experiments were conducted at a spinning rate of 12.5 kHz. The ^13^C Larmor frequency was 100.64 MHz, and tetramethylsilane (TMS) was used as the external reference. A CP/MAS pulse sequence with a contact time of 2 ms and a relaxation delay of 2 s was applied. Membrane morphology, including surface and cross-sectional structure, was characterized by scanning electron microscopy (SEM) using a Zeiss AURIGA Laser microscope (Carl Zeiss SMT, Oberkochen, Germany) at an accelerating voltage of 30 kV and a beam current of 2 pA. Surface topography was further assessed via atomic force microscopy (AFM) using an NT-MDT NTegra Maximus instrument (NT-MDT Spectrum Instruments, Moscow, Russia) operating in tapping mode with silicon cantilevers (15 N/m).

The hydrophilic and hydrophobic surface characteristics of both cross-linked dense and supported membranes were evaluated through water contact angle measurements using the sessile drop method on an LK-1 goniometer (NPK Open Science, Krasnogorsk, Russia). The DropShape software (version 2.5, KRUESS GmbH, Hamburg, Germany) was used for data analysis. Measurements were performed on both sides of dense membranes, while for supported membranes, only the selective dense layer side was analyzed. Thermochemical stability was assessed through thermogravimetric analysis (TGA) using a TG 209 F1 Libra analyzer (Netzsch, Selb, Germany), performed under an argon atmosphere with a controlled heating rate of 10 °C/min.

## 4. Conclusions

Efficient bio-derived mixed matrix membranes (MMMs) based on carboxymethyl cellulose (CMC) modified with carboxylated graphene oxide (GOCOOH) were successfully developed and evaluated for sustainable water purification through pervaporation and nanofiltration techniques.

To identify the most effective modifier concentration, CMC membranes incorporating varying GOCOOH contents (ranging from 3 to 7 wt.%) were tested for pervaporation dehydration of isopropanol–water mixtures (containing 12–30 wt.% water). The addition of GOCOOH to the CMC led to increased permeation flux while preserving excellent water selectivity in the permeate (99.9 wt.%). These performance gains were linked to morphological modifications observed through SEM and AFM, resulting from hydrogen bonding interactions between CMC and GOCOOH (validated by FTIR), as well as enhanced surface hydrophilicity (supported by contact angle analysis). Among all variants, the membrane containing 5 wt.% GOCOOH (referred to as CMC-5) showed the best performance in terms of permeation flux and was selected as the optimal composition. To enhance membrane durability and performance under high water concentration conditions, both unmodified and this GOCOOH-modified membranes were cross-linked using glutaraldehyde (GA). The resulting cross-linked membranes demonstrated improved selectivity and operational stability throughout the wide feed composition range (up to 90 wt.% water). In particular, the CMC-5 membrane cross-linked with GA (CMC-5^GA^) exhibited not only an increased permeation flux but also an improved water concentration in the permeate (>99.9 wt.%). These enhancements were credited to the combined effect of GOCOOH incorporation and GA cross-linking, which collectively disrupted CMC crystallinity and enhanced structural uniformity within the membrane matrix (as evidenced by NMR analysis).

For practical application potential, the CMC-based thin selective layer was deposited onto a porous polyamide (PA) substrate to form supported membranes. Among these, the cross-linked supported membrane CMC-5/PA^GA^ exhibited the best performance in isopropanol dehydration, delivering superior stability in dilute aqueous systems, with a permeation flux over 2.5 and consistent water selectivity above 99.9 wt.%, outperforming the pristine dense CMC membrane. To assess multifunctionality, this supported membrane was also tested in nanofiltration of aqueous solutions with anionic dyes, heavy metals, and inorganic salts. The membrane achieved high rejection rates—exceeding 98.5% for dyes and 99.8% for heavy metal ions—demonstrating strong potential as a sustainable and adaptable material for environmentally friendly wastewater treatment. This dual application is conditioned by the properties of the CMC material and modifier, which are characterized by their hydrophilic nature, abundant functional groups that enable cross-linking, and affinity for water. The combination of these applications will facilitate the treatment of complex streams, particularly organic solvents or wastewaters containing dissolved ions and larger molecules. Future research on these membranes could focus on examining their performance under harsh conditions (e.g., high temperatures, organic solvents, high contaminant concentrations), testing real wastewater streams from various sources, and developing integrated membrane processes for potential industrial applications.

## Figures and Tables

**Table 1:** Designations of the developed CMC-based membranes.

Membrane	Type	Content of GOCOOH, wt.%	Cross-Linking Method
CMC	dense	-	-
CMC-3	dense	3	-
CMC-5	dense	5	-
CMC-7	dense	7	-
CMC^GA^	dense	-	1 wt.% GA and 0.5 wt.% H_2_SO_4_
CMC-5^GA^	dense	5	1 wt.% GA and 0.5 wt.% H_2_SO_4_
CMC/PA^GA^	supported	-	1 wt.% GA and 0.5 wt.% H_2_SO_4_
CMC-5/PA^GA^	supported	5	1 wt.% GA and 0.5 wt.% H_2_SO_4_

**Table 2:** The average (Ra) and root mean square (Rq) roughness and water contact angle of developed CMC-based membranes.

Membrane	Surface Parameters		Water Contact Angle, °
	Ra, nm	Rq, nm	
CMC	5.5	6.9	55 ± 3
CMC-5	8.3	12.8	52 ± 2
CMC^GA^	106.7	134.3	49 ± 3
CMC-5^GA^	124.3	180.5	44 ± 4
CMC/PA^GA^	6.0	9.2	48 ± 4
CMC-5/PA^GA^	14.8	21.6	42 ± 3

## Data Availability

Data are contained within the article and Supplementary Materials.

## Supplementary Materials

The following supporting information can be downloaded at: https://www.mdpi.com/article/10.3390/molecules30183751/s1, Figure S1: Elemental analysis via EDS for the (a) CMC, (b) CMC-5 and (c) CMC-5^GA^ membranes; Figure S2: TG curves of dense CMC-based membranes; Figure S3: Preparation scheme of dense CMC-based membranes; Figure S4: Preparation scheme of supported CMC-based membranes; Figure S5: Schematic representation of the pervaporation setup; Figure S6: Schematic representation of the nanofiltration setup; Table S1: Main characteristics of the tested dyes.
