# Supplementary material for: Membranes from Carboxymethyl Cellulose/Carboxylated Graphene Oxide for Sustainable Water Treatment by Pervaporation and Nanofiltration
Source: Molecules. 2025 Sep 15;30(18):3751. doi: 10.3390/molecules30183751 (PMC12472858; doi:10.3390/molecules30183751)
Supplement: Supplementary file 1 [file molecules-30-03751-s001.zip › molecules-3851821-supplementary.pdf]

molecules

MDPI

Membranes from Carboxymethyl Cellulose/Carboxylated Graphene Oxide for Sustainable Water Treatment by Pervaporation and Nanofiltration

Mariia Dmitrenko [1,*], Olga Mikhailovskaya [1], Anna Kuzminova [1], Anton Mazur [1], Rongxin Su [2] and Anastasia Penkova [1,*]

[1] St. Petersburg State University, 7/9 Universitetskaya nab., St. Petersburg 199034, Russia; st113220@student.spbu.ru (O.M.); a.kuzminova@spbu.ru (A.K.); a.mazur@spbu.ru (A.M.)

[2] State Key Laboratory of Chemical Engineering, School of Chemical Engineering and Technology, Tianjin University, Tianjin 300072, China; surx@tju.edu.cn

* Correspondence: m.dmitrienko@spbu.ru (M.D.); a.penkova@spbu.ru (A.P.); Tel.: +7-(812)-363-60-00 (ext. 3367) (M.D. & A.P.)

The chemical analysis of membranes using energy dispersive X-ray spectroscopy (EDS) was carried out to determine elemental composition and a uniform distribution of the carbon signal throughout the membrane (Figure S1).

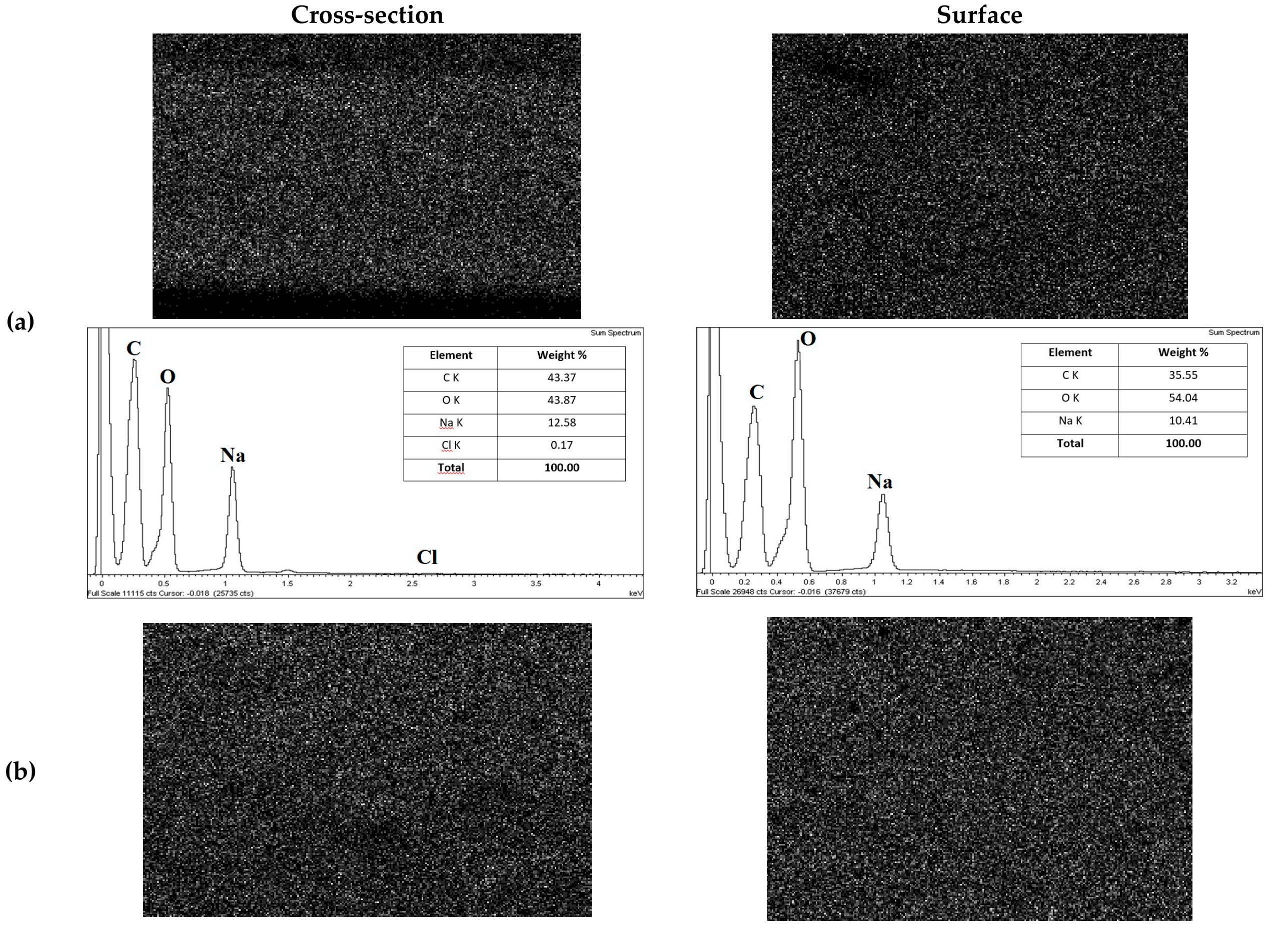

(c)

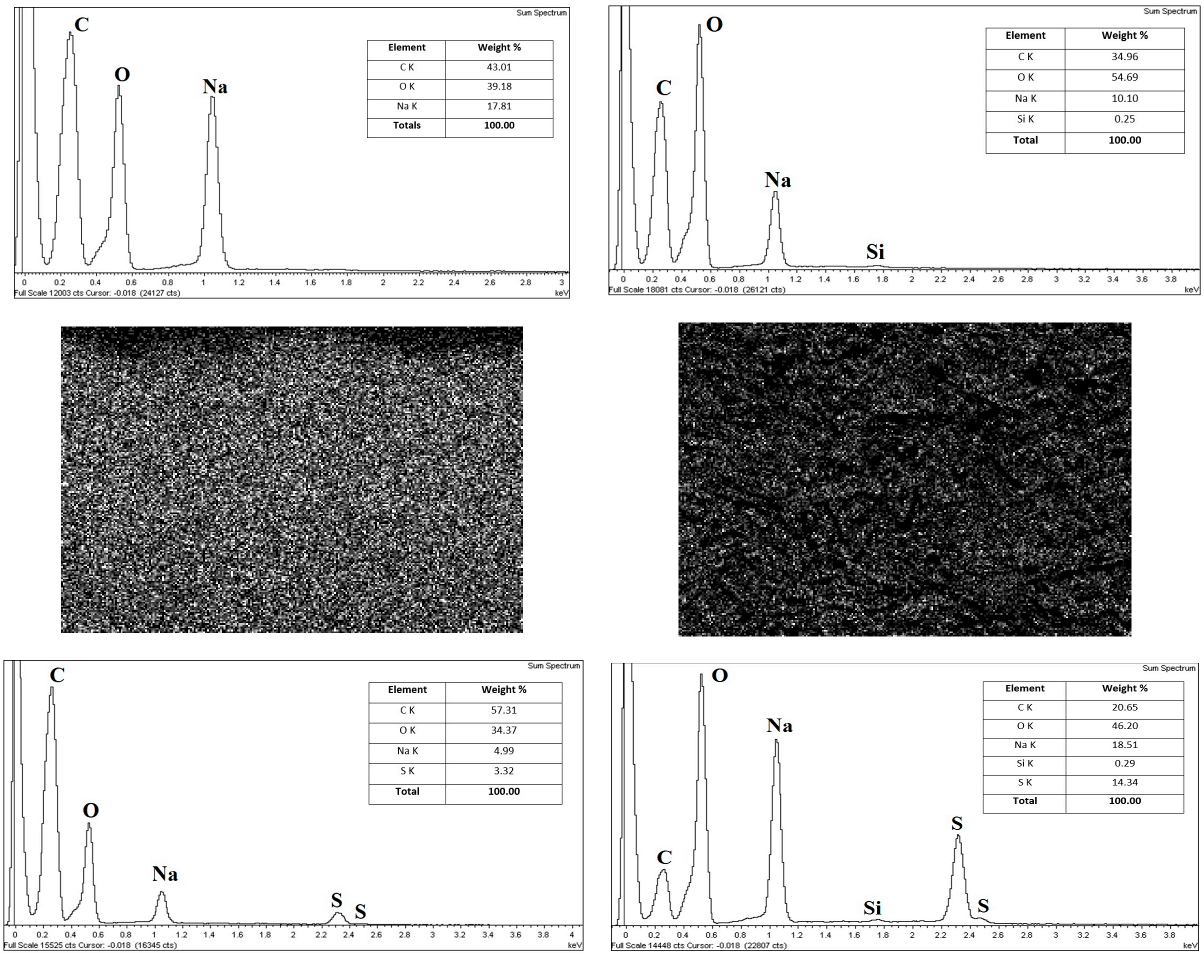

Figure S1. Elemental analysis via EDS for the **(a)** CMC, **(b)** CMC-5 and **(c)** CMC-5^{GA} membranes.

The elemental mapping confirmed a uniform distribution of the elemental signal throughout the membranes. The presence of Si was due to contamination from equipment during analysis, while the presence of S for the CMC-5^{GA} membrane was due to the cross-linking taking place in the presence of acid as a catalyst. Additionally, elemental mapping obtained via EDS confirm the uniform distribution of other elements within the membranes, indicating successful homogenization.

The thermal stability of the developed dense membranes was assessed by TGA. The corresponding thermograms (TG) are shown in Figure S2.

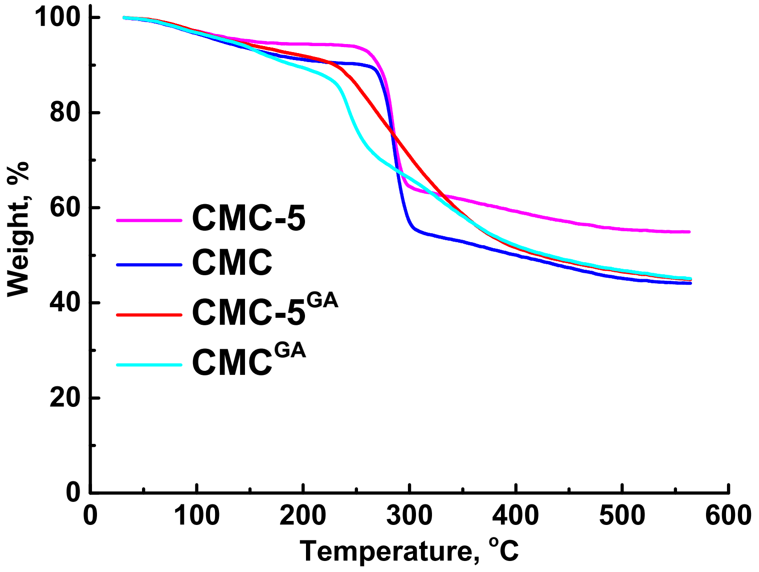

Figure S2. TG curves of dense CMC-based membranes.

Preparation scheme of dense CMC-based membranes is presented in Figure S3.

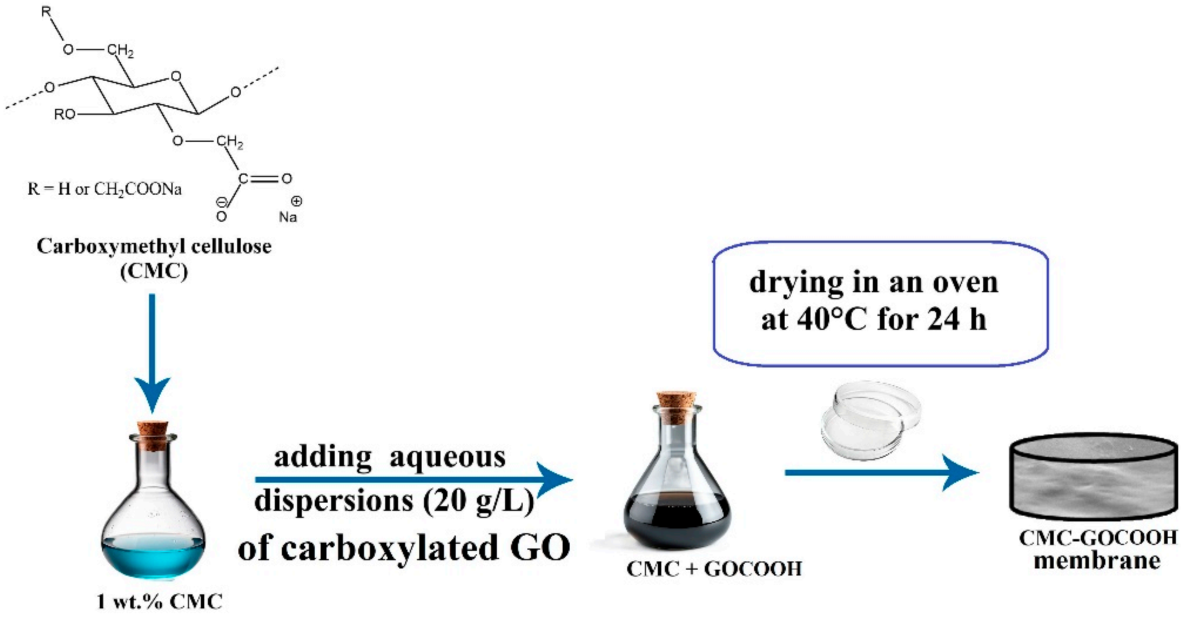

Figure S3. Preparation scheme of dense CMC-based membranes.

Preparation scheme of supported CMC-based membranes is presented in Figure S4.

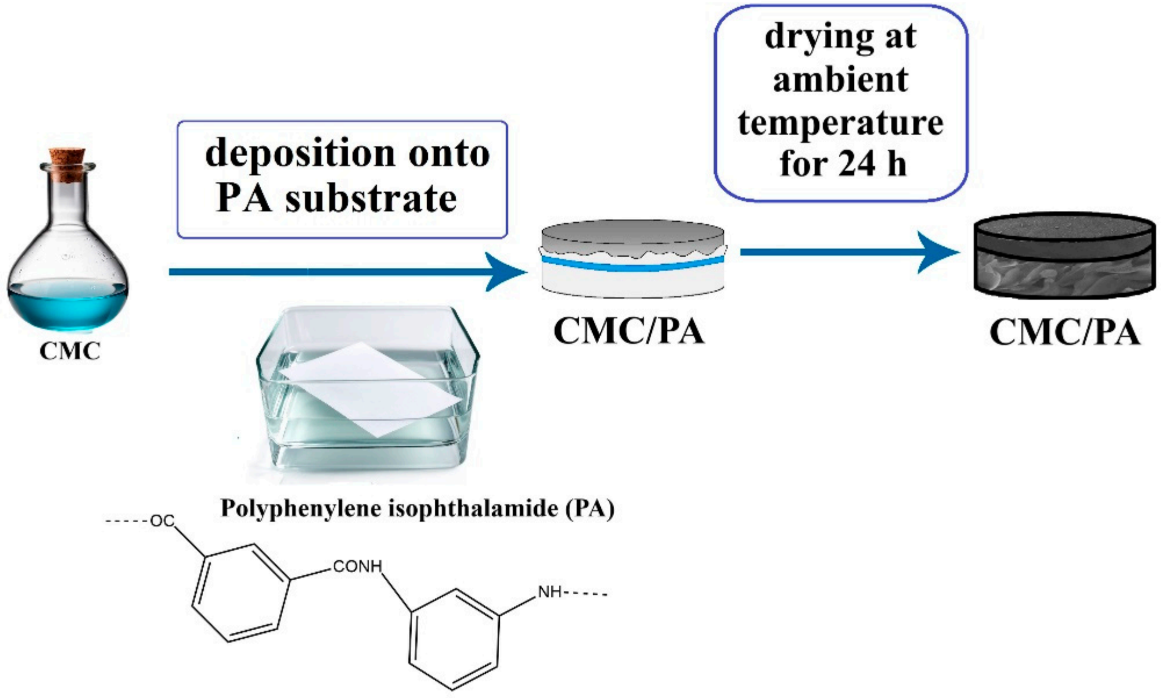

Figure S4. Preparation scheme of supported CMC-based membranes.

Schematic representation of the pervaporation setup is presented in Figure S5.

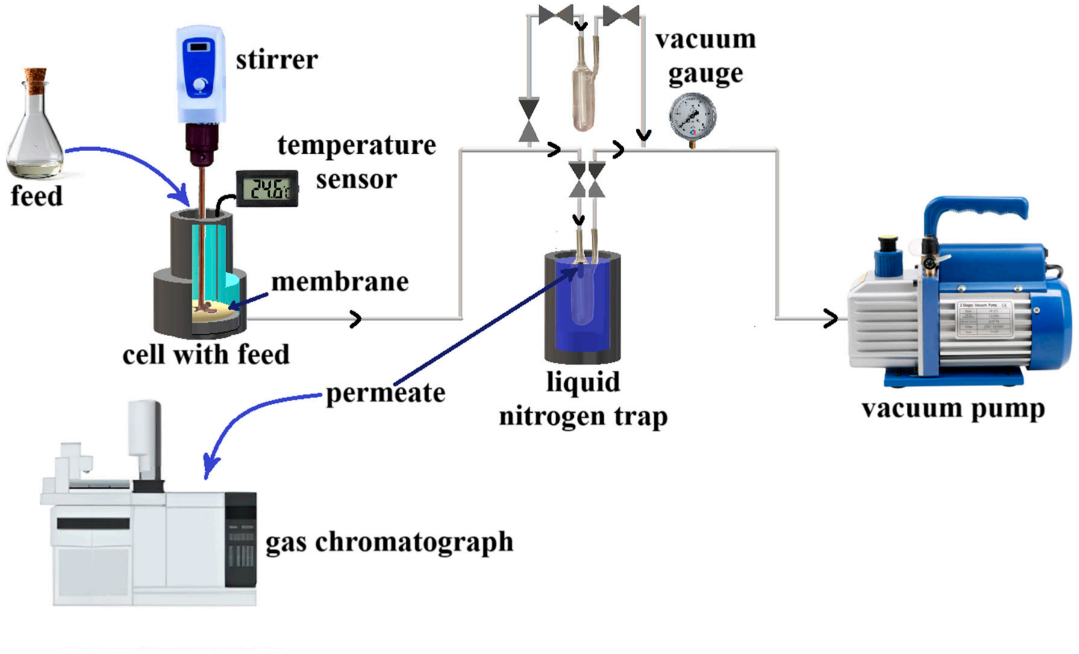

Figure S5. Schematic representation of the pervaporation setup.

Schematic representation of the nanofiltration setup is presented in Figure S6.

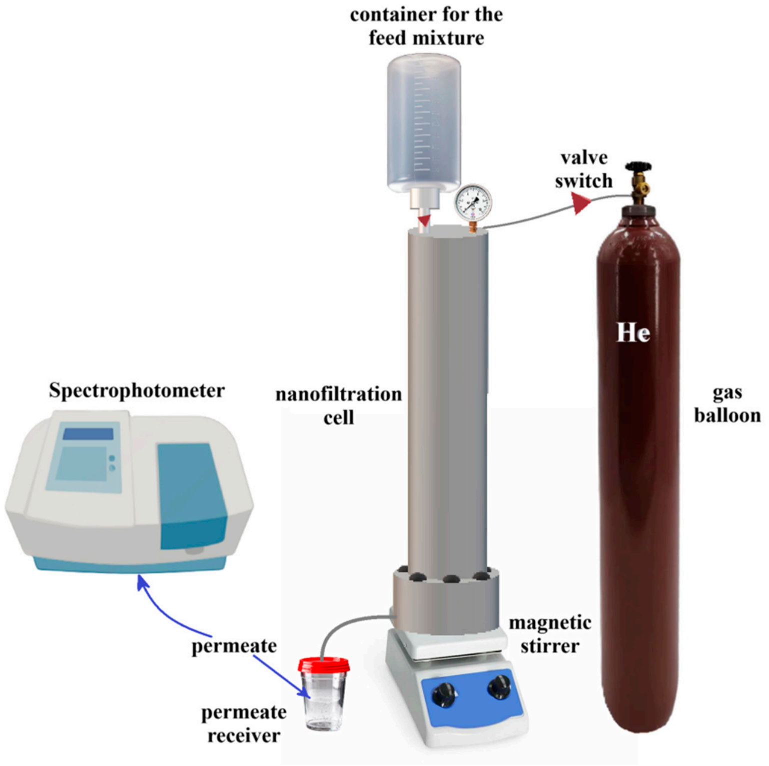

Figure S6. Schematic representation of the nanofiltration setup.

Table S1. Main characteristics of the tested dyes.

Dye	Abbreviation	Structure	Linear Formula	Molar Mass (g/mol)
Sunset yellow	SY	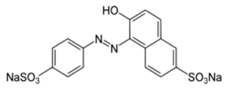	$C_{16}H_{10}N_2Na_2O_7S_2$	452.37
Congo red	CR	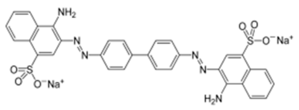	$C_{32}H_{22}N_6Na_2O_6S_2$	696.67
Alphazurine	AZ	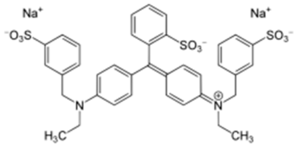	$C_{37}H_{35}N_2NaO_6S_2$	792.85
